# A Digital Cancer Ecosystem to Deliver Health and Psychosocial Education as Preventive Intervention

**DOI:** 10.3390/cancers14153724

**Published:** 2022-07-30

**Authors:** Laura Ciria-Suarez, Laura Costas, Aida Flix-Valle, Maria Serra-Blasco, Joan C. Medina, Cristian Ochoa-Arnedo

**Affiliations:** 1 eHealth ICOnnecta’t and Psycho-Oncology Services, Institut Català d’Oncologia, 08908 L’Hospitalet de Llobregat, Spain; laura.ciria.suarez@gmail.com (L.C.-S.); aflixv@iconcologia.net (A.F.-V.); mariaserrab@iconcologia.net (M.S.-B.); jmedina1@uoc.edu (J.C.M.); 2 Cancer Epidemiology Research Programme, IDIBELL, Institut Català d’Oncologia, 08908 L’Hospitalet de Llobregat, Spain; lcostas@iconcologia.net; 3 Consortium for Biomedical Research in Epidemiology and Public Health (CIBERESP), 28029 Madrid, Spain; 4 Psycho-Oncology and Digital Health, Health Services Research in Cancer, Institut d’Investigació Biomèdica de Bellvitge (IDIBELL), 08908 L’Hospitalet del Llobregat, Spain; 5 Department of Clinical Psychology and Psychobiology, Universitat de Barcelona, 08035 Barcelona, Spain; 6 Department of Psychology, Universitat Abat Oliba CEU, 08022 Barcelona, Spain; 7 Department of Psychology and Education Sciences, Universitat Oberta de Catalunya, 08018 Barcelona, Spain

**Keywords:** breast cancer, educational strategy, internet-based intervention, cancer survivors, stepped-care, psychosocial intervention

## Abstract

**Simple Summary:**

With the recent increase in survival rates of breast cancer patients, it is of key importance to also improve their life quality. Disinformation regarding illness is one of the major stress sources for patients with breast cancer. The present study aimed to study the educational section of the digital ecosystem ICOnnecta’t, analyzing which health information areas are most relevant for breast cancer patients. The fact that patients mostly consulted emotional and medical audiovisual material within the first three months after diagnosis underlines the need to create significant health-related content and deliver it to patients shortly after diagnosis. Those preventive interventions are essential to avoid the deterioration of emotional distress, which in turn has been shown to influence, not only life quality, but also patient survival.

**Abstract:**

Health education and psychosocial interventions prevent emotional distress, and the latter has been shown to have an impact on survival. In turn, digital health education interventions may help promote equity by reaching a higher number of cancer patients, both because they avoid journeys to the hospital, by and having a better efficiency. A total of 234 women recently diagnosed with breast cancer in a comprehensive cancer center used the digital ecosystem ICOnnecta’t from March 2019 to March 2021. ICOnnecta’t consists of four care levels, provided to patients according to their level of distress. The second level of this intervention consists of an educational campus, which was analyzed to track users’ interests and their information-seeking behavior. Overall, 99 out of 234 women (42.3%) used the educational campus. There were no significant differences in sociodemographic and clinical variables between the campus users and non-users. Among users, the median number of resources utilized per user was four (interquartile range: 2–9). Emotional and medical resources were the contents most frequently viewed and the audiovisual format the most consulted (*p* < 0.01). Resources were used mainly within the first three months from enrolment. Users who were guided to visit the virtual campus were more active than spontaneous users. Offering an early holistic health educational platform inside a digital cancer ecosystem, with health professionals involved, can reach more patients, promoting equity in the access of cancer information and prevention, from the very beginning of the disease.

## 1. Introduction

Breast cancer (BC) is the most common cancer worldwide [1], being the leading cause of death in women aged 20–50 years [2]. In 2020, there were around 2.3 million new BC diagnoses and 685,000 BC deaths worldwide [3]. Despite this increased incidence, survival has improved in the recent years, mainly as a consequence of therapeutic advances and early diagnosis [4]. These data evidence that prevention and screening are key to dealing with this critical health and social problem [5].

Cancer is a life-threatening disease that affects all spheres of life, impacting on emotional, mental, and behavioral reactions [6]. In effect, a diagnosis triggers a complex set of implications, such as coping with the new situation, managing physical symptoms, re-adjusting the relationship with the family, and dealing with the existential dimension of the illness. Moreover, such impacts can be observed both in the short and medium term after diagnosis [7]. The management of the illness and treatment side effects increases stress and uncertainty; anxiety, pessimism, and depression; physical pain, discomfort, and social isolation; which overall diminish the quality of life of BC patients [8,9,10].

To date, psychosocial interventions, including supportive information, social support, and cognitive therapy, have been proven to have positive effects in patients [11]. In fact, increasing cancer knowledge, self-management skills, and self-efficacy in communication could also improve the quality of life of BC patients [9]. Emotional distress prevention through such strategies has become a key challenge in the cancer journey, as evidence shows their impact on patient’s health and the possibility to reduce it with proper psychosocial interventions [12]. In the same line, receiving a psychosocial intervention during oncological treatment can improve a patient’s health [13]. Indeed, a recent meta-analysis by Oh et al. (2016) showed that psychosocial interventions during the early phase of cancer were associated with a reduction in mortality in 41% of cases [14]. A crucial part of such psychosocial interventions consists of health literacy [15], which is the degree to which individuals can obtain, process, and understand basic health information. Such information is required to engage in healthier decisions, such as adherence to cancer screening programs [16] or exercising after breast cancer treatment [17].

In order to address the psychosocial burden observed in BC patients, innovative interventions are needed, especially taking into account the most vulnerable populations [18]. To this aim, eHealth (i.e., healthcare services provided electronically) is a growing field, which is transforming health promotion and healthcare delivery, and which provides a new opportunity to reach and engage with communities [19]. Interventions through eHealth appear to be a solution to meet the psychosocial needs of BC patients, helping them to improve their health literacy, such as their cancer information management skills and emotional functioning, thus improving their quality of life [8]. eHealth has the potential to reach a large number of people; however, there are barriers to digital technology engagement, such as limited digital and traditional health literacy [20]. To assure equity, eHealth programs must address the variability of digital health literacy of users (e.g., disadvantaged groups, older patients) [20], as well as capturing the perspectives from all sociocultural communities [19].

ICOnnecta’t is a stepped eHealth ecosystem led from a public, monographic, oncological hospital located in Southern Europe and supported by Horizon 2020, through the European Institute of Innovation and Technology, which pursues the deployment of new digital tools in cancer care. The objective of this program applied to BC patients is to support them by, first, monitoring their symptoms and assessing psychosocial risk through a mobile application, and, if needed, offering them educational information and psychosocial care [21,22]. Therefore, ICOnnecta’t is structured in four stepped levels: (1) screening and monitoring, (2) education resources, (3) peer-support community, and (4) online-group psychotherapy. The program has been developed together with BC patients and professionals, with the focus on creating a platform adapted to the specific circumstances of this target population. This digital program allows monitoring patients and offering resources to patients who otherwise could not have access to them, either due to constraints related to face-to-face visits (e.g., mobility or economic difficulties) or to hospital capacity (e.g., limited staff availability). Indeed, the ecosystem was created to democratize psychosocial care and health education in cancer. Recently, ICOnnecta’t has proven to be a successful eHealth tool to monitor symptoms and psychosocial needs, facilitating access to guided early interventions [22]. Digital psychosocial education resources may promote equity in the access of knowledge, and therefore result in an increased quality of life of BC patients

In ICOnnecta’t, BC patients can access the second care level, education resources, both autonomously (directly from the app) or prescribed by a professional when they detect distress during the screening and monitoring (first care level of the ecosystem). Teaching strategies for patient education, such as audio and videotapes, and written materials, have been shown to increase knowledge, decrease anxiety, and increase satisfaction [23]. Therefore, the present study aimed to describe and assess the use of the educational section of ICOnnecta’t (virtual campus, level 2) in a sample of recently diagnosed BC patients during the first two years of the ecosystem’s implementation.

## 2. Materials and Methods

### 2.1. Study Design

This study follows a quasi-experimental, single-group, longitudinal design.

It was developed according to the 1964 Helsinki declaration and its later amendments and was approved by the Clinical Research Ethics Committee of the leading institution on 25 October 2018 (PR343/18). 

### 2.2. Recruitment and Participants

BC patients were recruited from the Catalan Institute of Oncology (ICO), a public and monographic center specialized in cancer, located in north-eastern Spain. This institute is made up of several facilities distributed in different locations, being the reference oncologic center for more than 40% of the adult population of Catalonia. ICO belongs to The Spanish National Healthcare System, which guarantees universal coverage and free healthcare access to all Spanish nationals, regardless of economic situation or participation in the social security network.

Healthcare professionals informed about the study to all BC patients recently diagnosed, and those interested were referred to the ICOnnecta’t service to arrange a visit. Afterwards, ICOnnecta’t personnel explained and clarified doubts about the program, checked eligibility criteria, invited the participant to sign the informed consent, guided them to install the digital ecosystem on their smartphones, and gave them basic management training to use it. At this point, participants were in the first care level of the program (i.e., screening and monitoring).

Participants recruited from 15 March 2019 (when the first patient accessed it) to 14 March 2021 were selected, so the first two years of the virtual campus data were analyzed. To allow sufficient longitudinal follow-up data, the first year of each participant was analyzed. Eligibility criteria were (1) adults (≥18 years), (2) diagnosed with a first episode of BC in the previous 3 months, (3) internet access and user-level skills, and (4) fluent in Catalan or Spanish. Exclusion criteria were: (1) major depressive disorder, psychosis, or substance abuse; (2) autolytic ideation; or (3) impaired cognition. 

### 2.3. ICOnnecta’t Intervention

ICOnnecta’t is an eHealth program addressed to cancer patients, to offer them a digital intervention through an app. It is organized into four levels of care, offered progressively, according to patients’ psychosocial needs. All patients join the program in the first level and they retain access to the previous levels if they step up [22,24].

Level 1: This consists of a screening and monitoring risk assessment system. Patients fill in periodical psychosocial questionnaires (reviewed by psychologists), while physical symptoms are monitored by nurses. Patients receive automatic health advice for their symptoms from the app, and tailored messages or (video)calls from health professionals, if needed. Similarly, if moderate or high emotional distress is identified, a psychologist offers the patient a videoconference to explore their needs and propose they access the second level of care, the educational platform.

Level 2: The second level consists of a wide variety of educational resources, through a virtual campus. Patients can access it directly from the ICOnnecta’t app, either spontaneously or guided by health professionals. The latter case occurs when distress is identified in level 1 and they are referred to level 2, as aforementioned. All education resources were selected and co-created with BC patients and health professionals. This article focuses on this second level of ICOnnecta’t, and therefore it is further described below. 

Level 3: This is a social community guided by health professionals, where BC patients can, in an anonymous way, share their experiences and ask questions about their needs to other patients. This community is structured by mirroring the campus co-created topics.

Level 4: This consists of a psychotherapy group offered by a specialized clinical psychologist. It is conducted through videoconference and is structured in eight weekly 90-min sessions, with a positive psychology approach [25].

Satisfaction with ICOnnecta’t and its usability as perceived by the participants were assessed three weeks after registration in the program with a 0–10 VAS. No clear interpretation bands were found in the literature. Therefore, we reported the scores ≥5 as satisfaction/usability approved by patients.

### 2.4. Description of Level 2, the Virtual Campus

The development of the educational platform (virtual campus) for patients was co-created in different steps, involving both BC patients and cancer health professionals. There was an expert patient involved throughout the process, who gave support in content development. This patient, a BC survivor and pedagogue, had been part of a support group with other patients, where she collected a varied range of experiences of survivors.

Before creating the content, an exploratory study in BC, of the factors involved in the use and sharing of internet information with health professionals, was developed [26]. In this study, two focus groups were held with 13 BC patients, and a questionnaire was administered to 186 BC patients afterwards. Through that questionnaire, the use and psychological impact of searching for information on the Internet was assessed. Similarly, focus groups were also held with 8 health professionals (i.e., psycho-oncologist, nurses, oncologist, radiotherapist, medical radiologist, and gynecologist), who provided guidance regarding the most demanded information in consultations. Afterwards, a questionnaire was administered to 59 health professionals about their perception of the use and psychological impact of searching for information online on patients. Finally, within the framework of hospital dissemination activities, some meetings were held with patients, where their feedback on future developments was collected.

The knowledge generated through all these procedures led to a virtual campus organized in 6 different thematic areas, where patients can consult various types of resources. On its homepage, participants can find a presentation of the space, describing the campus; highlighting the information reliability, veracity, and rigorousness; and an endorsement by professionals. In the same page, patients have access to the information of all the professionals involved in the project.

The 6 thematic areas are the following:

1. The first thematic area is called “my emotions”. Here, patients can find videos, closed-questionnaires, experience-questions, and text information for the emotional impact of cancer, sadness, fear, and irritability.
2. The second area is related to the disease and treatments. Resources in this area are videos, closed-questionnaires, and experience-questions about surgery, chemotherapy, radiotherapy, brachytherapy, breast reconstruction, and cancer-associated thrombosis.
3. The third area is about “my personal relationships”, where information about children (both communication with them and their emotional experience), about the conspiracy of silence and on how to face the visits with the oncologist. Resources in this area are again videos, closed-questionnaires, experience-questions, and text information.
4. The fourth area is related to the body, concretely about body image, hereditary cancer, and sexuality. Resources in this area are videos, closed-questionnaires, experience-questions, text information, and one infographic.
5. The fifth area is about a healthy lifestyle, where information about nutrition and rest (sleep and recommendations for insomnia) is featured. In this area, all type of formats can be found.
6. The sixth area is related to daily life and activities. This last section contains information about the taboo of cancer. Resources in this area are videos, closed-questionnaires, experience-questions, and text information.

These resources have been expanded over time, we restricted the present analyses to those resources that were available before the first patient was recruited, to avoid a temporal bias.

### 2.5. Statistical Analyses

Descriptive analyses were performed using a Chi-square test for categorical parameters and non-parametric Kruskal–Wallis test for non-normally distributed continuous variables. Median and interquartile ranges were used as measures of central tendency and dispersion. All analyses were conducted using Stata version 16.0 (Statacorp, Texas, US), and graphs were performed using R version 4.1.2. (R Core Team, Vienna, Austria).

## 3. Results

### 3.1. Participant Characteristics

During the first two years of ICOnnecta’t, 348 BC patients were enrolled in the program. Among these, 234 participants were considered “users of the first level”, since they completed at least one psychosocial questionnaire or physical symptom in Level 1 of the program. Of these 234 “users of the first level”, 99 consulted at least one resource from Level 2 of the program, the virtual campus (considered “users” herein). The sociodemographic characteristics and clinical stage of the users (if they utilized at least one resource of the virtual campus) and non-users (if they did not utilize any resources), are shown in Table 1. Regarding the age, the mean age of the participants was 51.61 (SD = 8.78), the median was 51 (IQR = 46–58), and the age ranged from 27 to 76. For users (*n* = 99), mean age was 50.35 (SD = 7.93), median was 49 (IQR = 45–56), and the age ranged from 30 to 72. For non-users (*n* = 135), mean age was 52.53 (SD = 9.27), median was 52 (IQR = 46–60), and age ranged from 27 to 76.

The average satisfaction level with the platform among the 134 participants who completed this measure was 5.72 (SD = 3.37). Up to 65.67% reported being satisfied. In turn, the mean platform usability perceived by the 178 participants who completed this measure was 7.74 (SD = 2.98), with 79.09% of them reporting the ecosystem as easy to use.

### 3.2. Resource Utilization

When the ecosystem was launched for BC patients, the campus had 66 educational resources. These resources had different formats (videos, closed questions, open questions, text, and infographics) and dealt with different topics (medical, emotional management, healthy lifestyle, social management, physical appearance, and daily life).

Among users, the median number of resources viewed was four (interquartile range: 2–9). Medical and psychological resources were the type of contents most frequently viewed, more than others such as healthy lifestyle, social management, physical appearance, and daily life (*p* < 0.01). The audiovisual content was the most consulted format, followed by texts, while infographics and questions were less consulted (median proportion of 13%, 7%, 0%, and 0% of the utilized resources, respectively, *p* < 0.01). Within the different topics, the most viewed thematic area was “my emotions” (median = 6% of viewed resources), followed by the disease and treatment area (median = 5% of viewed resources, *p*-value < 0.01 for types of content). Among the users of the campus, the median viewed four educational resources, and the 85.9% watched at least one video, see Table 2.

### 3.3. Patterns of Utilization: Guided vs. Spontaneous Use

As exposed above, once patients have logged in ICOnnecta’t app, they can access the virtual campus in two different ways. Guided use involves a health professional actively recommending the campus (level 2) in one of the first level interactions. On the other hand, spontaneous use was defined as autonomous use without any health professional indication. Of the 99 campus’ users (BC patients who consulted at least one resource in the virtual campus) 50 were guided and 49 made a spontaneous use. From the 135 patients who were non-users, 28 BC patients were guided but, in the end, they did not use any of the resources.

Guided users consulted a median of seven educational resources per person (IQR = 2–10), while spontaneous users consulted a median of three (IQR = 2–8). There were statistical differences between spontaneous use and guided use by type of format (*p* = 0.035 and 0.001 for text and infographics, respectively) and content (*p* = 0.033 and 0.048 for medical and social management, respectively), see Table 3.

There were no significant differences in utilization by tumor stage (*p* = 0.536).

### 3.4. Patterns of Utilization over Time

**Figure 1:**
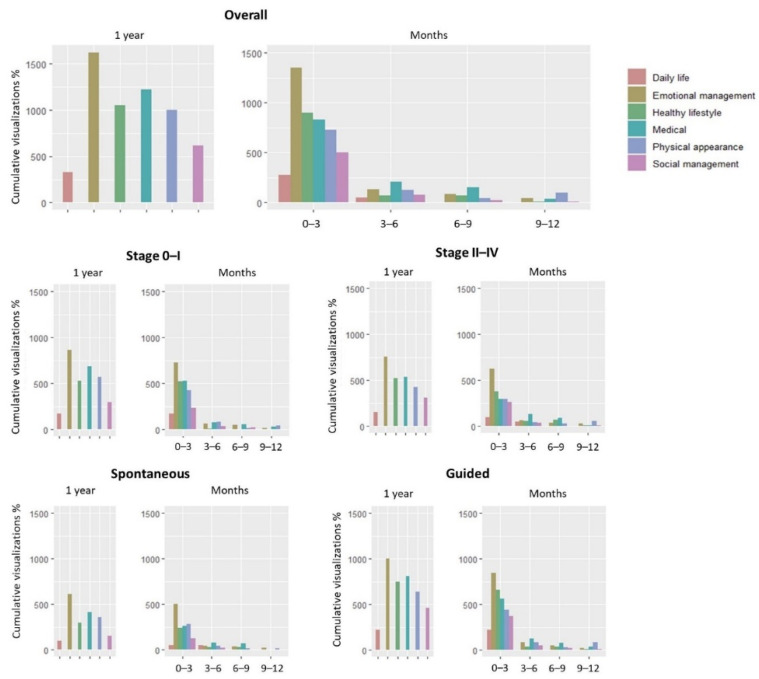
Utilization of resources over time.

The highest number of educational resource consultations occurred during the first 3 months after BC patients joined the ICOnnecta’t program (Figure 1). During the first months, the most viewed were the emotional aspects. The consultation of all resources decreased over time, for all topics. Notably, there was a small change in this trend between 9–12 months for physical appearance. Resources related to medical aspects decreased less markedly than the rest, being the most viewed between 3 and 9 months.

## 4. Discussion

ICOnnecta’t is an eHealth ecosystem to deliver preventive education and psychosocial care in cancer. This research describes the behavior of BC patients regarding the educational section of ICOnnecta’t during the first two years of its implementation. No significant differences were found for sociodemographic and clinical variables (age, marital status, occupational status, year of diagnosis, and clinical stage) between the patients who accessed the virtual campus and consulted any resource (users), versus those who did not view any resource (non-users), showing that the variables with an impact in SES and related with equity do not influence the access/use of eHealth care programs in the BC journey. Previous literature described that there are differences when accessing information through electronic devices in BC patients. In particular, the degree of digitization depended on age, education, and household size; although, the presence of internet access, internet use, and the availability of mobile devices for internet use increased from 2012 to 2020 [27]. It is recommended to offer instruction and support services, especially for middle-aged and older patients to boost patient engagement [28]. But the eHealth behavior is not only influenced by SES variables, as points out the study of Faber et al. [29], which details the importance of eHealth interventions being aligned with the person’s attitude. Such research, which investigates the eHealth attitudes of people living in a neighborhood with low SES, identify two general attitudes. The first one, which represented approximately half of the sample, was optimistically engaged, involved light-heartedness toward health, loyalty toward healthcare, and eagerness to adopt eHealth. These results are in line with other studies that also show that participants with low SES can engage in eHealth interventions [30,31,32,33]. The second attitude, embodied roughly a quarter of the sample, were doubtfully disadvantaged, feeling hesitance toward eHealth adoption. They propose, among others, that eHealth intervention should suit the day-to-day of the person, have personal communication, and adapt it to literacy level and life situation. In this regard, there are other studies that detail the importance of coaches in studies with participants with low SES [30,33,34]. In our sample, we have not seen differences by sociodemographic variables between users and non-users, of the educational campus (level 2). It is important to point that the relationship of the patient with the ICOnnecta’t eHealth platform is always mediated by a healthcare professional. Therefore, offering an education platform integrated into a more comprehensive online digital health program (ICOnnecta’t), offered personally and with installation and problem-solving support provided to each patient, probably facilitates adherence to the platform and promotes equity in its access.

Regarding the preferred format of consulted materials, videos were the most accessed, while text, infographics or questions were less consulted. This finding is in line with the fact that the second most visited website in the world is YouTube, which contains 60% of all videos of Internet and may be used as a health educational resource [35,36]. Studies that investigated the quality of YouTube videos about several cancer types (colorectal, prostate and breast) found poor quality and accuracy in health content [35,36,37]. The virtual campus from ICOnnecta’t constitutes a user-friendly content-provider offering information created by hospital professionals, thus guaranteeing validity and fostering prevention in disease management.

Respecting the type of content, emotional and medical resources were the most frequently visualized, over healthy lifestyle, social management, physical appearance and daily life. Hongru Lu et al. [38], in the synthesis about information needs of BC patients, detailed that 94% of the studies reported that patients were concerned about treatment information, such as intervention procedures, side effects and preoperative procedures. In general, disease-focused information are the most engaging type of information [39,40]. Hongru Lu et al. [38] also pointed out that doctors do not pay enough attention to the emotional pressure that patients feel during the diagnosis and treatment, remarking the need to address this kind of information. This finding agrees with the literature regarding the high rates (30–60%) of cancer-related distress after a BC diagnosis [7,41], and the few of them (fewer than 30%) receiving psychosocial care [41].

Regarding patterns of visualization, differences were observed between patients who were guided (those for whom a healthcare professional, having interacted with them at level 1, has proposed to consult a/some specific resources) versus those who accessed the virtual campus in a spontaneous way. Those guided visualized more resources than spontaneous users. Patients may sometimes feel that they are receiving too much information while, at some other times, they feel that the information is not enough; therefore, it is important to ensure that they receive a balanced information, in the appropriate format, and at the right time [42]. In order to reach such a challenging goal, a plausible approach is to tailor the information to each patient [43], and offering step-by-step guidance [44], which could favor the patient comfort to search the specific information needed. Thus, the strategy used in ICOnnecta’t, helps the patient receiving the information they lack, as a health professional also detects patients’ needs.

Regarding the utilizations over time, the highest number occurred during the first three months of joining ICOnnecta’t. As the literature remarks, patients value information early in the disease pathway [45]. After cancer diagnosis, patients’ information needs are broad; there is a significant level of adjustment [45] and they are interested in the diagnosis, treatments, side effects, finances, and strategies for coping with social and emotional aspects [46]. Furthermore, the moment of diagnosis is surrounded by a high emotional impact that entails an emotional whirlwind, which must be managed gradually [7]. This situation could explain the high number of emotional resource views, especially as a major need from the beginning. Healthy lifestyle information, such as nutrition and rest, were also resources viewed during these first months, which could imply the need of patients to take care of themselves and to feel active in their own recovery. BC patients are known to be one of the most active populations regarding their disease. As has been highlighted, medical information is one of the most important areas to consider [39,40], and we also saw that its access decreased more gradually, since many of the patients were receiving aggressive medical treatments during 3 to 9 months. Finally, it should be noted that the interest in physical appearance showed a small peak at 9–12 months, which could be explained by many factors. First of all, in many situations this timing coincides with breast reconstruction. Secondly, it is also the time when patients must prepare to return to “normality” [7]. Finally, physical concerns may appear once the most threating aspects of the illness are diminishing. It is important to note that patients can access this information from the beginning of their disease, offering prevention and offering a higher feeling of satisfaction [33].

When we detailed the views by stage (0–I vs. II–IV) and type of use (spontaneous vs. guided use), we found that most views were also made in the first 0–3 months (with emotional management resources the most consulted), and that the tendency was to progressively decrease over time in a similar way to the total views. In the case of the comparison between cancer stages 0-I and II-IV, we observed that in the 3–6 months period, the most prominent content for the II-IV stages was the medical content, but for those of lower stages, the physical aspect was slightly above the medical aspect in this period. In the later stages, physical appearance rebounded between 9–12 months. As stages 0–I do not receive chemotherapy, the active treatment (surgery and radiotherapy) is administered for 5–6 months, while more advanced stages usually have a duration of active treatment (surgery, chemotherapy, and radiotherapy) of about 1 year. In line with what we stated above, physical appearance is a content that is displayed in a more relevant way once the active treatments have finished.

There are some limitations that should lead to taking some results cautiously and serve as recommendations for further studies. The organization of the resources in the virtual campus could have affected their utilization, with the ones presented in the first locations being more viewed (first thematic area was “my emotions” and the second area was related with the disease and treatments). During the two first years of ICOnnecta’t implementation, some resources were added; this extension of resources could have interfered with the results of the utilization. Moreover, due to technical issues, the views were registered only the first time that the resource was consulted (not each time it was accessed). Finally, this study was carried out with a single group, of a small sample size; therefore, it would be more valuable to validate the information with a larger sample size and educational resources, as well as with a randomized and more controlled design. In any case, this study serves as a starting point for future studies, investigating the best content, format, and time to deliver health knowledge to BC patients. Similarly, we deem it crucial to estimate the cost-effectiveness of eHealth programs, in order to inform their future uptake by health providers. This aim is being pursued in a RCT, which is currently in the recruiting phase [24]. Once a program is developed, the main costs are related to the update and expansion of the educational resources, patient follow up from an educational psychologist, and maintenance of the technological platform. Such investment needs to be contemplated by providers interested in solutions such as ICOnnecta’t, which are foreseen to be surpassed by their advantages.

## 5. Conclusions

The findings of this study provide clear recommendations regarding the information about which BC patients are interested. Following the increasing interest in BC mobile apps that focus on secondary and tertiary prevention [47], the virtual campus integrated in the digital ecosystem ICOnnecta’t provides a secure environment with rigorous information that can be prescribed by professionals, to cover BC patients’ health-related information requirements. We would like to highlight that such requirements comprise, not only medical, but also emotional information, from the very beginning of the disease. In addition, the video format is the preferred way to receive health information, and patients are more likely to use it if prescribed by a health professional. Finally, the current findings show that patients’ information requirements change, depending on the moment of their cancer journey. By covering the lack of information frequently experienced by patients, we are promoting prevention from the very beginning of their disease.

## Figures and Tables

**Table 1:** Description of participants.

		Non-Users(*n* = 135)	Users(*n* = 99)	*p*-Value *
		***n*(%)**	***n*(%)**	
**Age (terciles)**				0.116
	**<47**	38(28.1)	34(34.3)	
	**47–55**	44(32.6)	39(39.4)	
	**56+**	53(39.3)	26(26.3)	
**Marital status**				0.107
	**Married or common-law partner**	97(71.9)	80(80.8)	
	**Separated or divorced**	15(11.1)	4(4.0)	
	**Single**	8(5.9)	8(8.1)	
	**Widow**	5(3.7)	1(1.0)	
	**missing**	10(7.4)	6(6.1)	
**Occupational status**				0.182
	**Active**	37(27.4)	33(33.3)	
	**Work leave**	51(37.8)	42(42.4)	
	**Occupational disability**	3(2.2)	0(0.0)	
	**Retired**	13(9.6)	4(4.0)	
	**Passive**	20(14.8)	11(11.1)	
	**missing**	11(8.1)	9(9.1)	
**Year of diagnosis**				0.573
	**2019**	80(59.3)	54(54.5)	
	**2020**	54(40.0)	43(43.4)	
	**2021**	1(0.7)	2(2.0)	
**Stage**				0.370
	**0-I**	68(50.4)	44(44.4)	
	**II-IV**	67(49.6)	55(55.6)	

* Chi squared, calculated without missing values, comparing participants and non-participants.

**Table 2:** Utilization of resources by type.

	Number of Resources	Total Number of Utilizations	Median Proportion of Utilized Resources, among Users (IQR)		Median Number of Resources Utilized per User, among Participants (IQR)		Women Who Have Utilized One Resource or More		
				*p*-Value *		*p*-Value *	*n*	% (among Users)	% (Overall)
**Type of format**									
**Videos**	16	323	13% (6–31%)	<0.01	2 (1–5)	<0.01	85	85.9%	36.3%
**Closed questions**	16	144	0% (0–13%)		0 (0–2)		41	41.4%	17.5%
**Open questions**	15	50	0% (0–0%)		0 (0–0)		23	23.2%	9.8%
**Text**	15	195	7% (0–20%)		1 (0–3)		66	66.7%	28.2%
**Infographics**	4	45	0% (0–25%)		0 (0–1)		31	31.3%	13.2%
**Type of content**									
**Medical**	20	245	5% (0–15%)	<0.01	1 (0–3)	<0.01	67	67.7%	28.6%
**Emotional management**	17	275	6% (0–24%)		1 (0–4)		53	53.5%	22.6%
**Healthy lifestyle**	10	105	0% (0–10%)		0 (0–1)		41	41.4%	17.5%
**Social management**	8	49	0% (0–0%)		0 (0–0)		22	22.2%	9.4%
**Physical appearance**	7	70	0% (0–14%)		0 (0–1)		41	41.4%	17.5%
**Daily life**	4	13	0% (0–0%)		0 (0–0)		8	8.1%	3.4%
**Total**	66	757	6% (3–14%)		4 (2–9)		99	100.0%	42.3%

* Non-parametric Kruskal–Wallis test, across type of format and content categories. IQR: Interquartile range.

**Table 3:** Use of resources among users by type of use: guided use vs. spontaneous use.

		Number of Available Resources		Median Proportion of Utilized Resources, among Users (IQR)		Median Number of Resources Utilized per User, among Users (IQR)	Women Who Utilized One Resource or More			
			Total Number of Utilizations		*p*-Value *		*n*	% (among Subgroup -Spontaneous or Guided-)	% (among Users)	% (Overall)
**ESPONTANEOUS USE (*n* = 49)**										
**Type of format**										
	**Videos**	16	125	13% (6–22%)	0.053	2 (1–4)	41	83.7%	41.4%	17.5%
	**Closed questions**	16	40	0% (0–6%)	0.256	0 (0–1)	19	38.8%	19.2%	8.1%
	**Open questions**	15	16	0% (0–0%)	0.783	0 (0–0)	11	22.4%	11.1%	4.7%
	**Text**	15	68	7% (0–13%)	0.035	1 (0–2)	29	59.2%	29.3%	12.4%
	**Infographics**	4	9	0% (0–0%)	0.001	0 (0–0)	8	16.3%	8.1%	3.4%
**Type of content**										
	**Medical**	20	83	5% (0–10%)	0.033	1 (0–2)	31	63.3%	31.3%	13.2%
	**Emotional management**	17	104	0% (0–18%)	0.096	0 (0–3)	22	44.9%	22.2%	9.4%
	**Healthy lifestyle**	10	30	0% (0–10%)	0.076	0 (0–1)	17	34.7%	17.2%	7.3%
	**Social management**	8	12	0% (0–0%)	0.048	0 (0–0)	7	14.3%	7.1%	3.0%
	**Physical appearance**	7	25	0% (0–14%)	0.170	0 (0–1)	18	36.7%	18.2%	7.7%
	**Daily life**	4	4	0% (0–0%)	0.450	0 (0–0)	3	6.1%	3.0%	1.3%
**Total**		66	258	5% (3–11%)	0.026	3 (2–8)	49	100.0%	49.5%	20.9%
**GUIDED USE (*n* = 50)**										
**Type of format**										
	**Videos**	16	198	19% (6–31%)		3 (1–5)	44	88.0%	44.4%	18.8%
	**Closed questions**	16	104	0% (0–13%)		0 (0–2)	22	44.0%	22.2%	9.4%
	**Open questions**	15	34	0% (0–2%)		0 (0–0)	12	24.0%	12.1%	5.1%
	**Text**	15	127	10% (0–20%)		2 (0–3)	37	74.0%	37.4%	15.8%
	**Infographics**	4	36	0% (0–25%)		0 (0–1)	23	46.0%	23.2%	9.8%
**Type of content**										
	**Medical**	20	162	13% (0–20%)		3 (0–4)	36	72.0%	36.4%	15.4%
	**Emotional management**	17	171	9% (0–25%)		2 (0–4)	31	62.0%	31.3%	13.2%
	**Healthy lifestyle**	10	75	0% (0–20%)		0 (0–2)	24	48.0%	24.2%	10.3%
	**Social management**	8	37	0% (0–13%)		0 (0–1)	15	30.0%	15.2%	6.4%
	**Physical appearance**	7	45	0% (0–18%)		0 (0–1)	23	46.0%	23.2%	9.8%
	**Daily life**	4	9	0% (0–0%)		0 (0–0)	5	10.0%	5.1%	2.1%
**Total**		66	499	10% (3–15%)		7 (2–10)	50	100.0%	50.5%	21.4%

* Non-parametric Kruskal–Wallis test, by guided use. IQR: Interquartile range.

## Data Availability

The anonymized datasets of this study may be obtained from the corresponding author upon reasonable request.
